# Refractive Error Correction With Glasses in Congenital Ocular Fundus Anomalies: A Retrospective Series of 18 Children With Different Disease Entities Followed Up for More Than 10 Years

**DOI:** 10.7759/cureus.94062

**Published:** 2025-10-07

**Authors:** Toshihiko Matsuo

**Affiliations:** 1 Department of Ophthalmology, Graduate School of Interdisciplinary Science and Engineering in Health Systems, Okayama University, Okayama University Hospital, Okayama, JPN

**Keywords:** charge syndrome, choroidal coloboma, coats disease, congenital eye anomalies, cycloplegic refraction, familial exudative vitreoretinopathy (fevr), full-correction glasses, morning glory disc anomaly, optic nerve coloboma, persistent fetal vasculature (pfv)

## Abstract

Objectives: Children with congenital anomalies of the posterior segment of the eye are in the process of visual development, and thus, their refractive errors should be detected by cycloplegic refraction testing to prescribe full-correction glasses, if required, and to help their visual acuity develop with growth. This study aimed to review refractive correction in children with congenital ocular fundus anomalies.

Methods: A retrospective review was conducted on 18 consecutive children (11 female and seven male children) who were diagnosed with ocular fundus anomalies and followed for 10 years or more by a single ophthalmologist at a referral-based hospital. The age at the initial visit ranged from 10 days after birth to 11 years, with a median of one year and four months, and the age at the last visit ranged from 10 to 32 years, with a median of 15 years. The follow-up periods ranged from 10 to 21 years at a median of 15 years.

Results: The diagnoses were familial exudative vitreoretinopathy (FEVR) in eight children, persistent fetal vasculature (PFV) in five, morning glory disc anomaly in two, optic nerve and choroidal coloboma (CHARGE syndrome) in two, and Coats disease in one. Full-correction glasses were prescribed in eight children, while the remaining 10 children did not wear glasses. Among nine children with the uncorrected visual acuity of 1.0 or better in one eye and the visual acuity in the other eye ranging from light perception to 0.01, eight children did not wear glasses, and one child wore glasses with hyperopic correction. The diagnoses in these nine children were PFV in five children, morning glory disc anomaly in two, FEVR in one, and Coats disease in one. In seven children who wore full-correction glasses, the best corrected visual acuity in the better eye ranged from 0.2 to 0.9 at a median of 0.5. In contrast, the visual acuity in the other eye ranged from light perception to 0.1 at a median of 0.03. The diagnoses of these seven children were FEVR in five children and CHARGE syndrome in two. The five children with FEVR showed myopic astigmatism in both eyes, while the two children with CHARGE syndrome showed hyperopic astigmatism in both eyes.

Conclusion: Children with unilateral eye anomalies such as PFV and morning glory disc anomaly did not wear glasses since their healthy eyes had good uncorrected visual acuity. In contrast, children with involvement of both eyes in FEVR and CHARGE syndrome wore full-correction glasses. Rough information regarding full-correction glasses in each category would help clinicians cope with rare congenital eye diseases. However, this conclusion is generally applicable to the standard practice of pediatric ophthalmology.

## Introduction

Congenital disorders are also referred to as congenital anomalies, malformations, or birth defects, and are defined as structural or functional anomalies that develop during intrauterine life. Both genetic and environmental factors play a role in the development of congenital disorders. In the management of congenital disorders, a key point to be kept in mind is the fact that children are in the process of physical and mental growth. In the field of ophthalmology, congenital disorders should be managed from the viewpoint of visual acuity and stereopsis, which are in the process of development, especially from birth to three years of age. Congenital eye diseases are rare and have a wide range of disease spectrum that affects different parts of the eyeball: anterior segment dysgenesis, such as Peters anomalies, persistent pupillary membrane, and congenital glaucoma [1], congenital cataract [2], ectopia lentis [3], vitreoretinopathy, optic disc, and choroidal coloboma [4,5]. The visual development is also influenced by congenital anomalies of the eye-supporting tissues, such as eyelid, ocular surface, and extraocular muscles: congenital blepharoptosis (eyelid ptosis) [6], ocular surface dermoid [7], and idiopathic superior oblique muscle palsy [8].

The posterior segment of the eye, also called the ocular fundus, consists of the retina, choroid, and optic nerve head (optic disc) and plays a major role in visual acuity and visual field. Well-known congenital diseases of the posterior segment are familial exudative vitreoretinopathy (FEVR) [9-12], persistent fetal vasculature (PFV; persistent hyperplastic primary vitreous) [10,13-16], and optic disc anomalies such as optic nerve coloboma and morning glory disc anomaly (morning glory syndrome) [17-21]. Congenital anomalies of the ocular fundus are typically considered inoperable, in contrast to ocular media anomalies such as congenital cataracts. As stated above, children with congenital anomalies of the posterior segment are in the process of visual development, and thus, their refractive errors should be detected through cycloplegic refraction testing. Full-correction glasses, if required, are prescribed to help their visual acuity develop in conjunction with growth [22-24]. In this study, the wearing of full-correction glasses, based on cycloplegic refraction testing, was reviewed in 18 consecutive patients with congenital anomalies of the posterior segment of the eye who were diagnosed and followed up by a single ophthalmologist in a single referral-based hospital. The author aimed to clarify whether refractive correction via cycloplegic testing could meaningfully contribute to visual development, particularly in cases with asymmetric or bilateral involvement of congenital eye diseases, which have often been overlooked due to their rarity and complexity, and where clinical decision-making is frequently inconsistent and poorly guided by the evidence.

## Materials and methods

A retrospective review was conducted on the medical records of 18 consecutive children with congenital anomalies of the posterior segment of the eye who were referred to the author and followed by him for 10 years or more between 2002 and 2024. This study was approved as a retrospective observational study by the Ethics Committee of the Graduate School of Medicine, Dentistry, and Pharmaceutical Sciences, Okayama University, and Okayama University Hospital (No. 2501-033, January 17, 2025). The exclusion criteria were 1) children whose visual acuity could not be measured due to mental delay or congenital eye diseases such as anophthalmos and microphthalmos, 2) children with optic nerve atrophy caused by perinatal hypoxia, and 3) children with premature birth or retinopathy of prematurity.

Retrieved from the medical records were the diagnosis, the age at the first visit and the last visit, best corrected visual acuity in decimals and refractive errors that were determined by cycloplegic refraction with 1% cyclopentolate at the first measurement and the last measurement, wearing of full-correction glasses based on cycloplegic refraction, history of ocular surgeries such as strabismus surgery, and other systemic features such as mental delay. During the data extraction process from medical records, a standardized form similar to Table 1 was used for data retrieval. The retrieved data were rechecked by the author on a different day during the validation step.

**Table 1:** Summary of 18 patients with congenital anomalies of the ocular fundus Refraction^*^ indicates cycloplegic refraction with 1% cyclopentolate D: diopters; c.: cylindrical; A: axis; PFV: persistent fetal vasculature; FEVR: familial exudative vitreoretinopathy; CF: counting fingers; HM: hand movement; LP: light perception; NLP: no light perception

Case/sex	Age at first visit	Follow-up period	Eye	Refraction^*^, visual acuity at first measurement	Age at first measurement	Clinical diagnosis	Other features	Glasses	Visual acuity at last measurement	Age at last measurement
1/Female	7 months	18 years	Right	0.6	3 years	None	None	No	1.5	18 years
			Left	CF		Morning glory disc anomaly			HM	
2/Male	1 year 10 months	20 years	Right	0.01	6 years (lost to follow-up after first visit)	Morning glory disc anomaly	Cataract surgery, vitrectomy, encircling, and gas tamponade for retinal detachment in the right eye at 6 years	No	HM (no retinal detachment)	21 years
			Left	1.0		None			1.2	
3/Female	11 years	21 years	Right	0.1	11 years	Retinal degeneration, dragged optic disc (FEVR)	Epilepsy and mental delay	No	0.1	32 years
			Left	HM		Falciform retinal detachment (FEVR), microcornea			HM	
4/Female	4 years	11 years	Right	0.05 × -3.5D c.-1.0DA180	4 years	Cataract, dragged optic disc, falciform retinal detachment (FEVR)	Nystagmus	Yes	0.04 × -5.0D	15 years
			Left	0.5 × -3.5D c.-1.0DA180		Macular hypoplasia (FEVR)			0.5 × -4.5D c.-2.5DA30	
5/Female	3 years	15 years	Right	0.1 × +3.0D	3 years	Choroidal coloboma	CHARGE syndrome (bilateral deafness, facial palsy, congenital heart disease)	Yes	0.7 (0.9 × +3.5D c.-1.0DA60	18 years
			Left	0.03 × +3.0D		Optic nerve and choroidal coloboma, esotropia			0.03 (0.03 × +6.0D	
6/Male	2 years 8 months	16 years	Right	HM	3 years	Retinal degeneration and vitreous opacity (FEVR), exotropia	Strabismus surgery at 14 years	No	0.01	18 years
			Left	1.0		Temporal peripheral retinal degeneration (FEVR)			1.5	
7/Female	3 years 8 months	16 years	Right	0.03	3 years	Coats disease	Panretinal photocoagulation in right eye at 3 years 9 months	No	LP	19 years
			Left	1.0		None			1.5	
8/Male	1 year 4 months	14 years	Right	0.5	3 years	None	Cataract in left eye, later	No	1.5	15 years
			Left	0.02		PFV			LP	
9/Female	5 months	15 years	Right	0.1	3 years	Dragged optic disc (FEVR)	Mild mental delay, small-for-date birth	Yes	0.1 (0.2 × +2.75D c.-3.0DA20	15 years
			Left	HM		PFV-like (FEVR), microcornea			HM	
10/Male	2 months	15 years	Right	0.3	3 years	None	Band-shaped keratoplasty in the left eye, later	No	1.2	15 years
			Left	Immeasurable		PFV, microphthalmia			LP	
11/Female	10 days	15 years	Right	0.9	3 years	None	Band-shaped keratoplasty in the left eye, later	No	1.5	15 years
			Left	Immeasurable		PFV, microphthalmia			NLP	
12/Female	2 months	15 years	Right	0.8	3 years	None	None	No	1.2	15 years
			Left	0.01		PFV, microphthalmia			CF	
13/Female	9 months	15 years	Right	0.01	3 years	PFV-like (FEVR), esotropia	Mild mental delay, nystagmus	No	0.03	15 years
			Left	0.03 × c.-1.5DA180		Retinal folds, dragged optic disc (FEVR)			0.09 × -2.5D c.-1.0DA180	
14/Male	1 month	15 years	Right	0.6	3 years	None	None	Yes	1.5 (1.5 × +3.0D)	15 years
			Left	Immeasurable		PFV			LP	
15/Male	1 year 6 months	14 years	Right	0.2 × -5.25D c.-2.5DA65	4 years	Dragged optic disc (FEVR)	Autism spectrum disorder	Yes	0.9 × -6.0D c.-2.0DA20	15 years
			Left	0.1 × -9.5D c.-1.5DA130		Dragged optic disc (FEVR)			0.7 × -10.0 c.-2.0DA165	
16/Male	5 months	12 years	Right	Immeasurable	3 years	Retinal folds, falciform retinal detachment (FEVR)	None	Yes	0.08 × -4.5D c.-2.0DA170	13 years
			Left	0.2 × -6.0D		Retinal degeneration (FEVR)			0.7 × -5.5D c.-1.0DA60	
17/Female	1 year 10 months	11 years	Right	Immeasurable	1 year 11 months	Optic nerve and choroidal coloboma, microphthalmia	CHARGE syndrome (bilateral deafness, persistent ductus arteriosus, right facial palsy)	Yes	HM × +11.25D c. -3.5DA180	12 years
			Left	+15.0D c.-5.0DA150		Optic nerve and choroidal coloboma, microphthalmia			0.2 × +5.75D c.-3.75DA70	
18/Female	2 months	10 years	Right	0.1 × -6.0D	3 years 6 months	Dragged optic disc (FEVR)	None	Yes	0.4 × -6.5D c.-4.5DA180	10 years
			Left	Immeasurable		PFV-like (FEVR), microcornea			LP	

Cycloplegic refraction testing was done at least once a year in the standard way: 1% cyclopentolate hydrochloride solution (Santen Pharmaceutical Co., Ltd, Osaka, Japan) was instilled in both eyes twice at an interval of five minutes, and autorefraction was done one hour after the first instillation. Autorefraction was done by either or both of the table-mounted autorefractor and the hand-held autorefractor. Under mydriasis induced by topical 1% cyclopentolate, fundus examinations and retinoscope refraction were performed in a standard procedure. The intraocular pressure was measured by a hand-held tonometer (iCare, Vantaa, Finland).

The visual acuity at a distance of 30 cm was tested using cards with a single Landolt C printed in different sizes (Single Landolt Test Cards For Near Point, Handaya, Tokyo, Japan) at a younger age. Then, after the learning experience in visual acuity testing, the visual acuity at a distance of 5 m was measured using cards with a single Landolt C printed in different sizes (Single Landolt Test Cards 5 m, Handaya). Finally, visual acuity was tested using vertical rows of Landolt C in different sizes, ranging from the largest size corresponding to 0.1 in decimal at the top to the smallest size corresponding to 2.0 at the bottom (International Standard Distant Test Chart, Landolt, 5 m, Handaya). Visual acuity worse than 0.1 was tested at a distance near a child using a card with a single Landolt-C in the size of 0.1 at a distance of 5 m. At each visit, every three or six months, the visual acuity in each eye was measured separately at 30 cm and 5 m to assess both near and distant vision. In data retrieval, distant visual acuity was chosen when both near and distant visual acuities could be measured. In case of the measurement only of near visual acuity at a younger age, the near visual acuity was retrieved.

## Results

**Figure 1:**
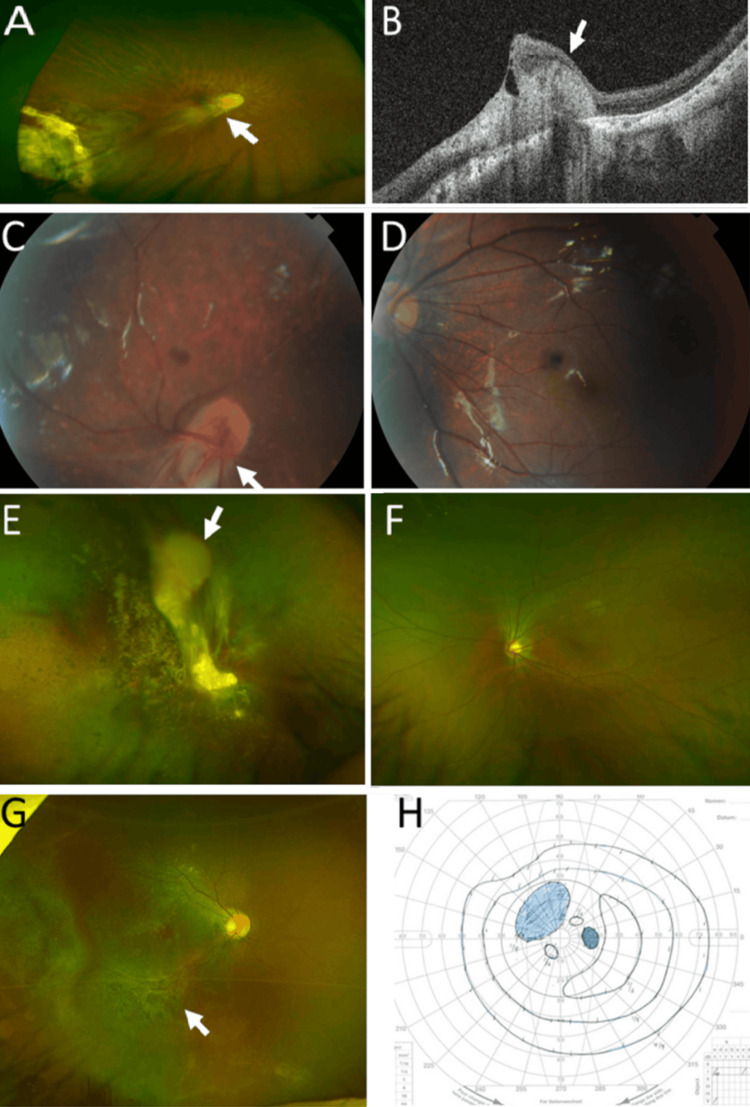
Familial exudative vitreoretinopathy Case 3: Right eye. Dragged optic disc (arrow) and temporal-side retinal degeneration in the widefield fundus photograph (A). Retinal fold (arrow) in the horizontal section image of optical coherence tomography at age 31 years (B). Case 4: Dragged optic disc (arrow) in right eye (C). Mild hypoplastic macula in the left eye at age eight years (D). Case 6: Retinal folds and retinal degeneration with vitreous opacity (arrow) in the right eye (E). Normal posterior-pole retina in the left eye at age 17 years (F). Case 9: Right eye. Retinal degeneration (arrow) in a widefield fundus photograph (G). Maintained peripheral visual field with inner scotoma by Goldmann perimetry in the right eye at age 15 years (H)

**Figure 2:**
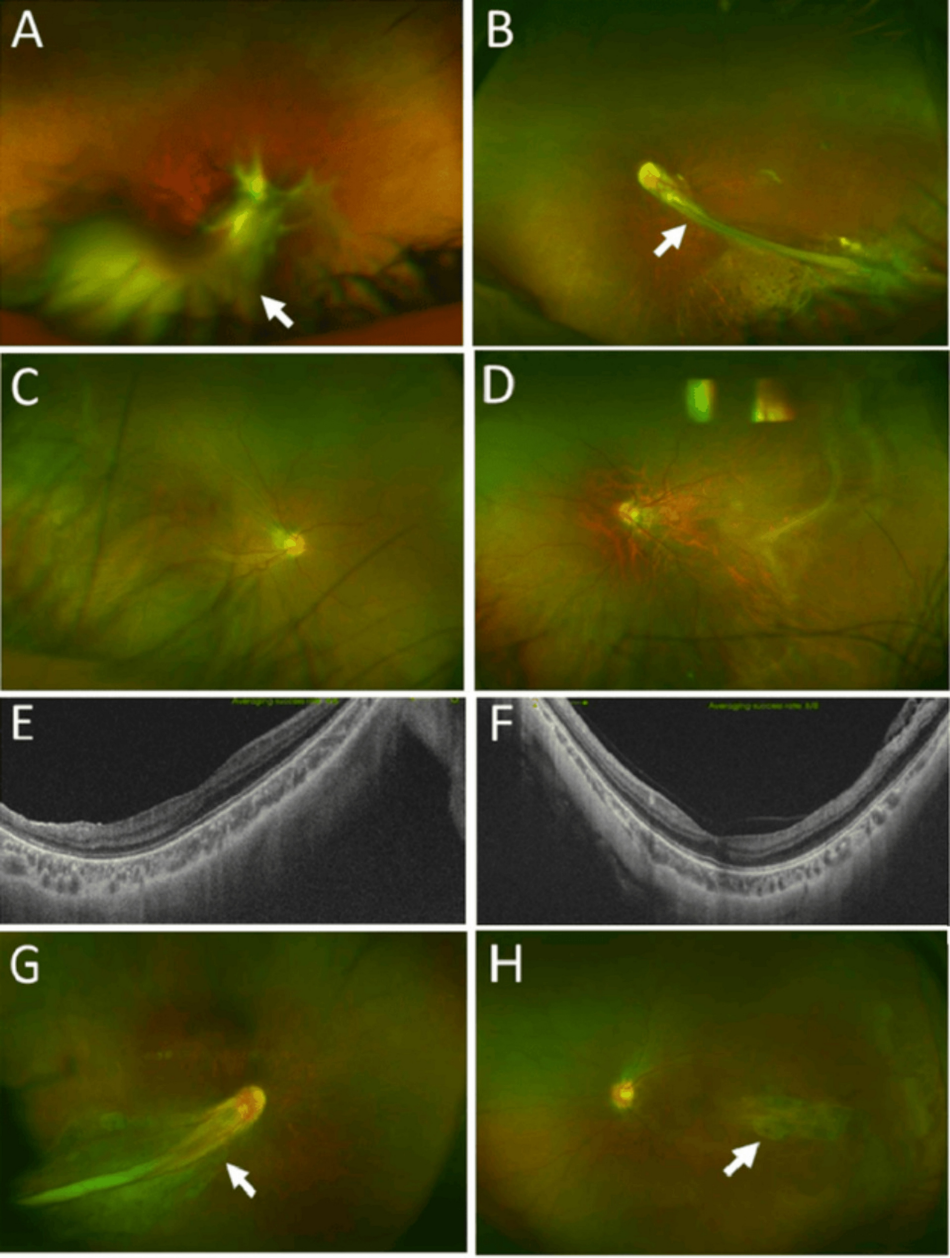
Familial exudative vitreoretinopathy Case 13: Widefield fundus photographs, showing fibrovascular tissue (arrow) arising from the optic disc in the right eye (A), and falciform retinal detachment (arrow) in the left eye (B) at age 15 years. Case 15: Widefield fundus photographs, showing dragged optic disc and temporal-side retinal degeneration in the right eye (C) and left eye (D) at age 14 years. Normal horizontal section images of optical coherence tomography in the right eye (E) and the left eye (F). Case 16: Widefield fundus photographs, showing falciform retinal detachment (arrow) in the right eye (G) and temporal-side retinal degeneration (arrow) in the left eye (H) at age 11 years

**Figure 3:**
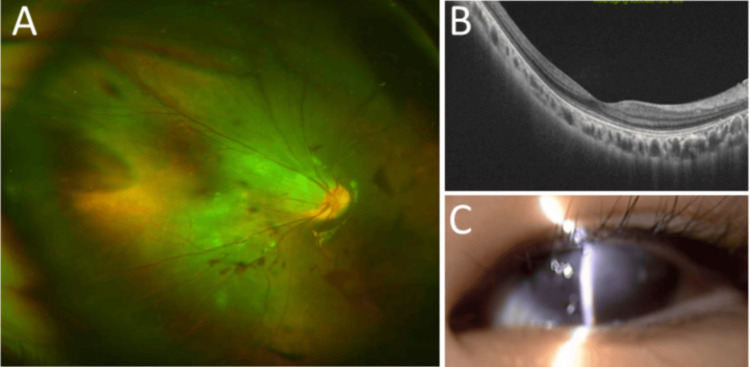
Familial exudative vitreoretinopathy Case 18: Dragged optic disc in a widefield fundus photograph (A) and a normal horizontal image of optical coherence tomography (B) in the right eye. Slit-lamp photograph (C), showing microphthalmia and a shallow anterior chamber in the left eye at age eight years

**Figure 4:**
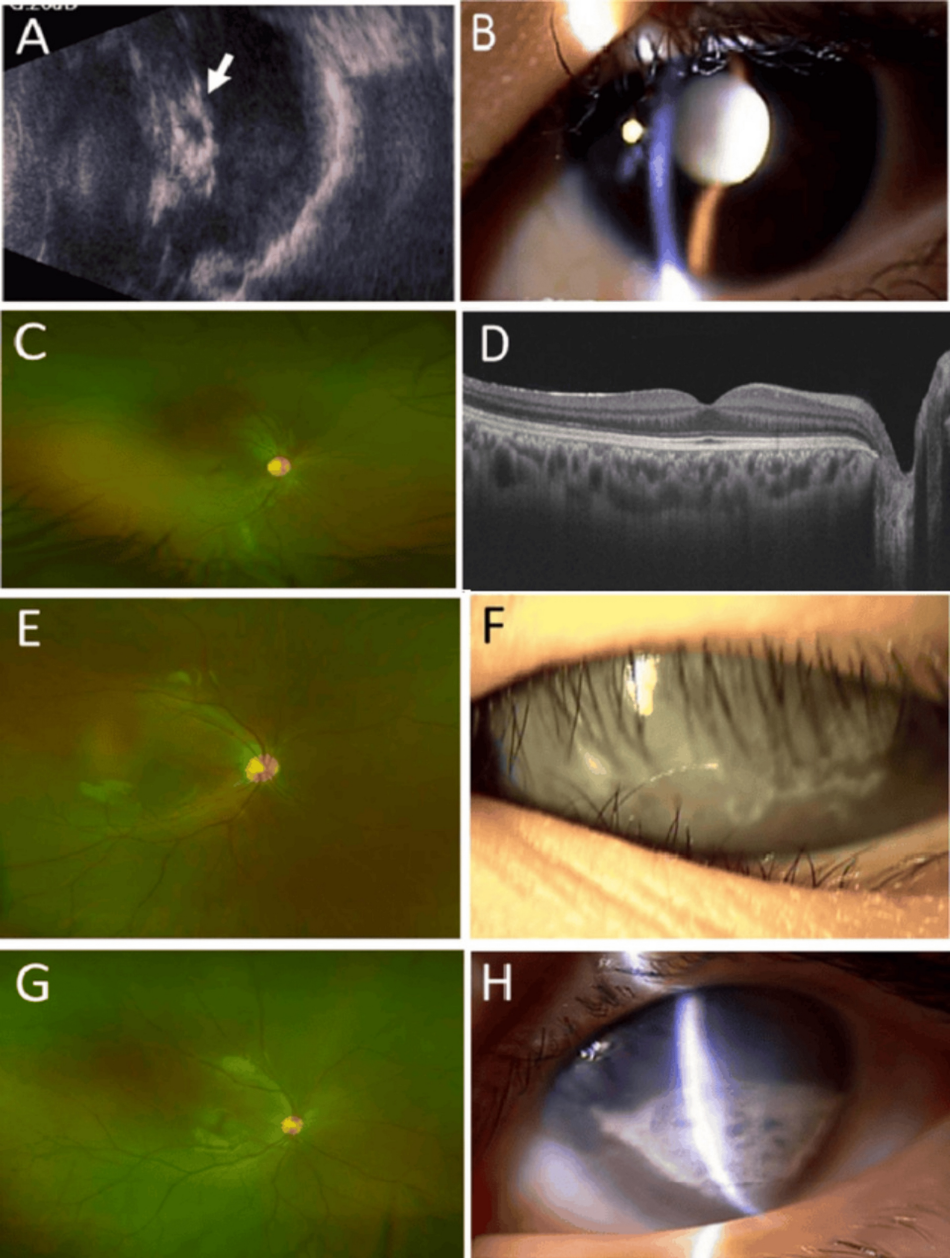
Persistent fetal vasculature Case 8: Vitreous opacity (arrow) in ultrasound examination (A) and mature cataract in slit-lamp photograph (B) in the left eye with persistent fetal vasculature at age 15 years. Normal widefield fundus photograph (C). Normal horizontal section image of optical coherence tomography in the right eye at age 15 years (D). Case 10: Normal widefield fundus photograph in right eye (E). Band-shaped keratopathy in the left eye with persistent fetal vasculature at age 12 years (F). Case 11: Normal widefield fundus photograph in the right eye (G). Band-shaped keratopathy and microphthalmia in the left eye with persistent fetal vasculature at age 12 years (H)

**Figure 5:**
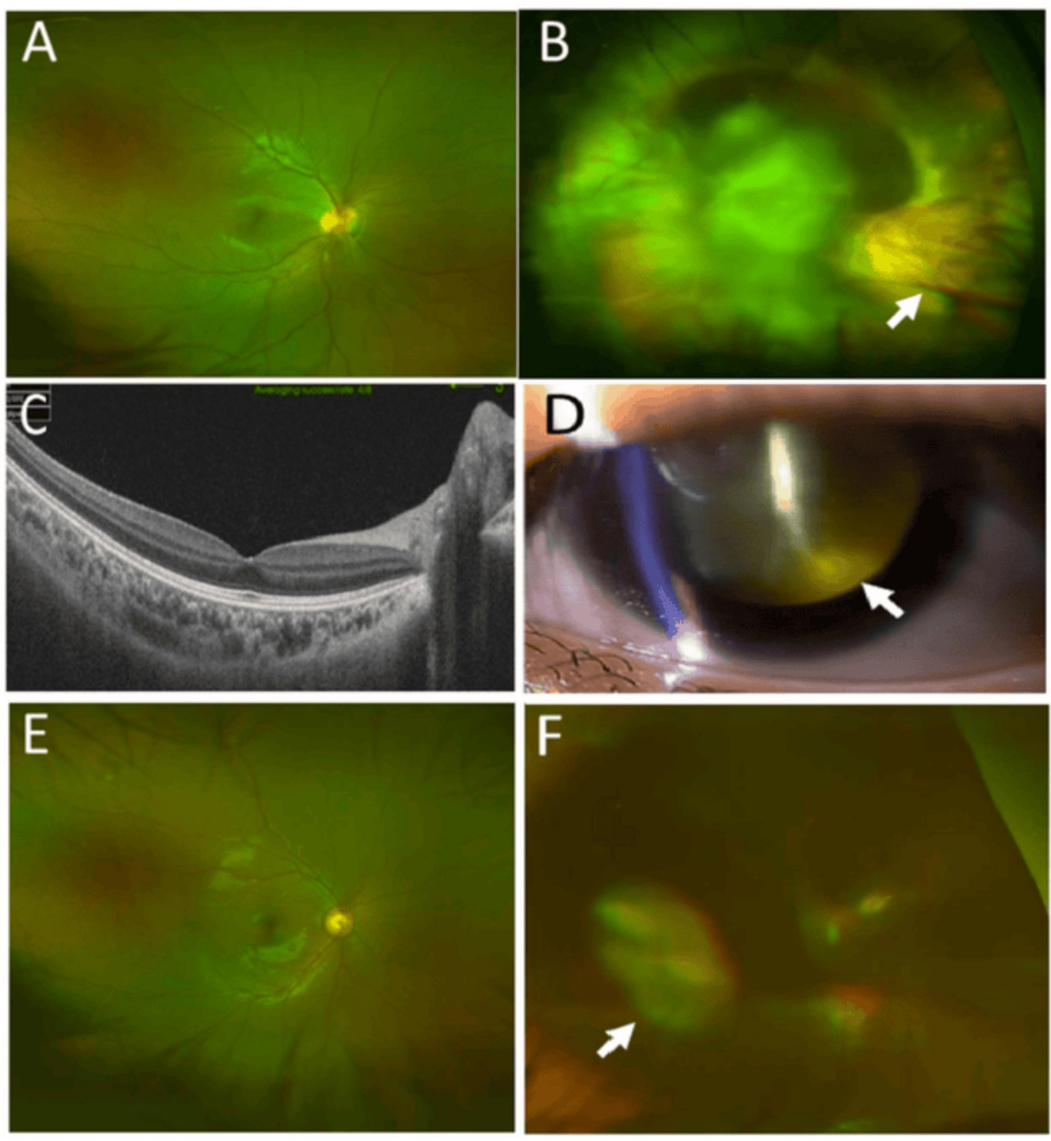
Persistent fetal vasculature Case 12: Widefield fundus photographs, showing normal fundus in the right eye (A) and fibrovascular tissue (arrow) arising from the optic disc in the left eye (B). Normal horizontal image of optical coherence tomography in the right eye (C). Slit-lamp photograph (D), showing fibrovascular tissue (arrow) posterior to the lens in the left eye at age 14 years. Case 14: Widefield fundus photographs, showing normal fundus in the right eye (E) and stalk-like vitreous opacity (arrow) in the left eye (F) at age 14 years

**Figure 6:**
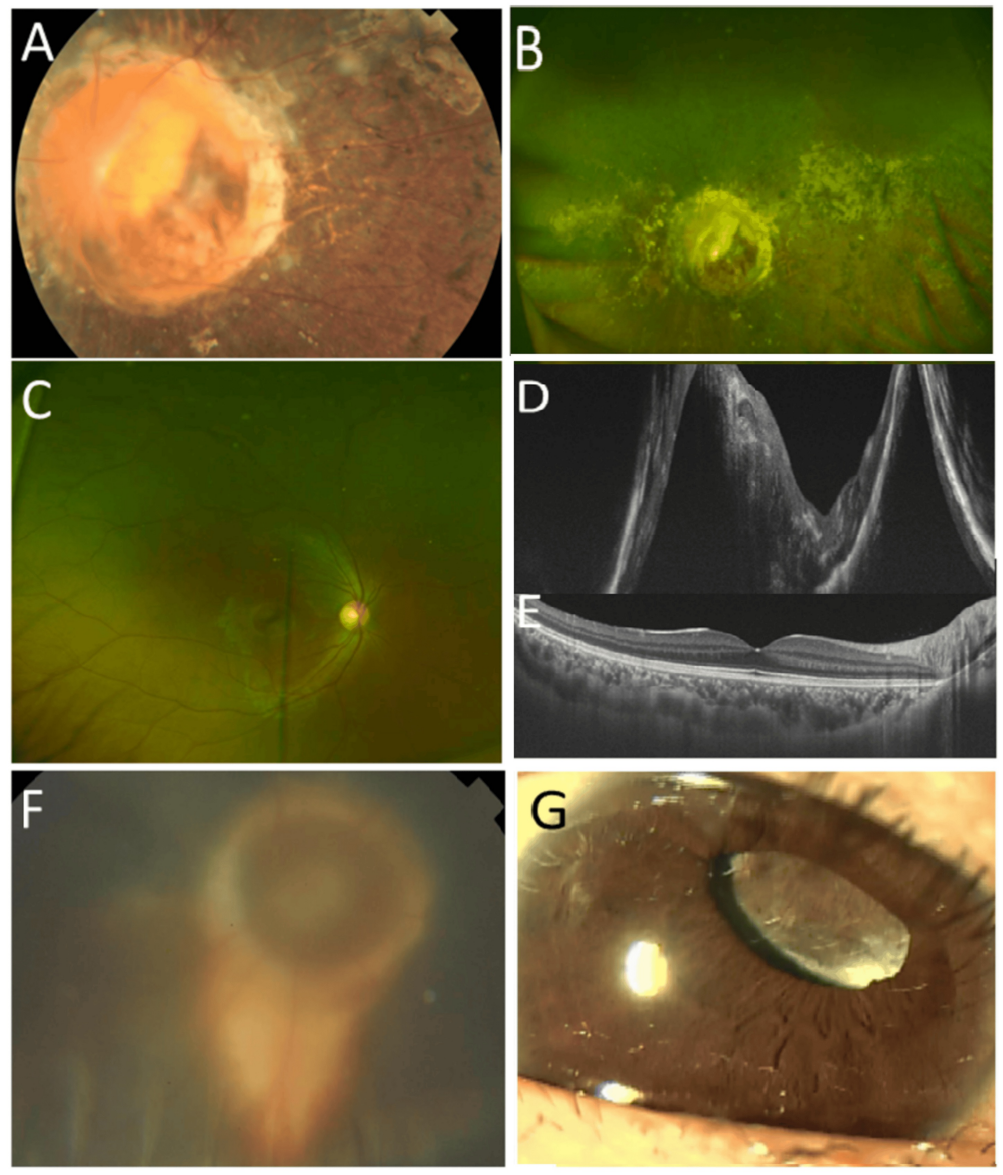
Morning glory disc anomaly Case 1: Left eye with morning glory disc anomaly: fundus photograph (A), widefield fundus photograph (B), horizontal section image of optical coherence tomography (D), in contrast with the normal right eye: widefield fundus photograph (C), horizontal section image of optical coherence tomography (E), at age 15 years. Case 2: Right eye with morning glory disc anomaly: fundus photograph three years after vitrectomy for retinal detachment at age six years (F). Slit-lamp photograph in the right eye with intraocular lens implantation at age 20 years (G)

**Figure 7:**
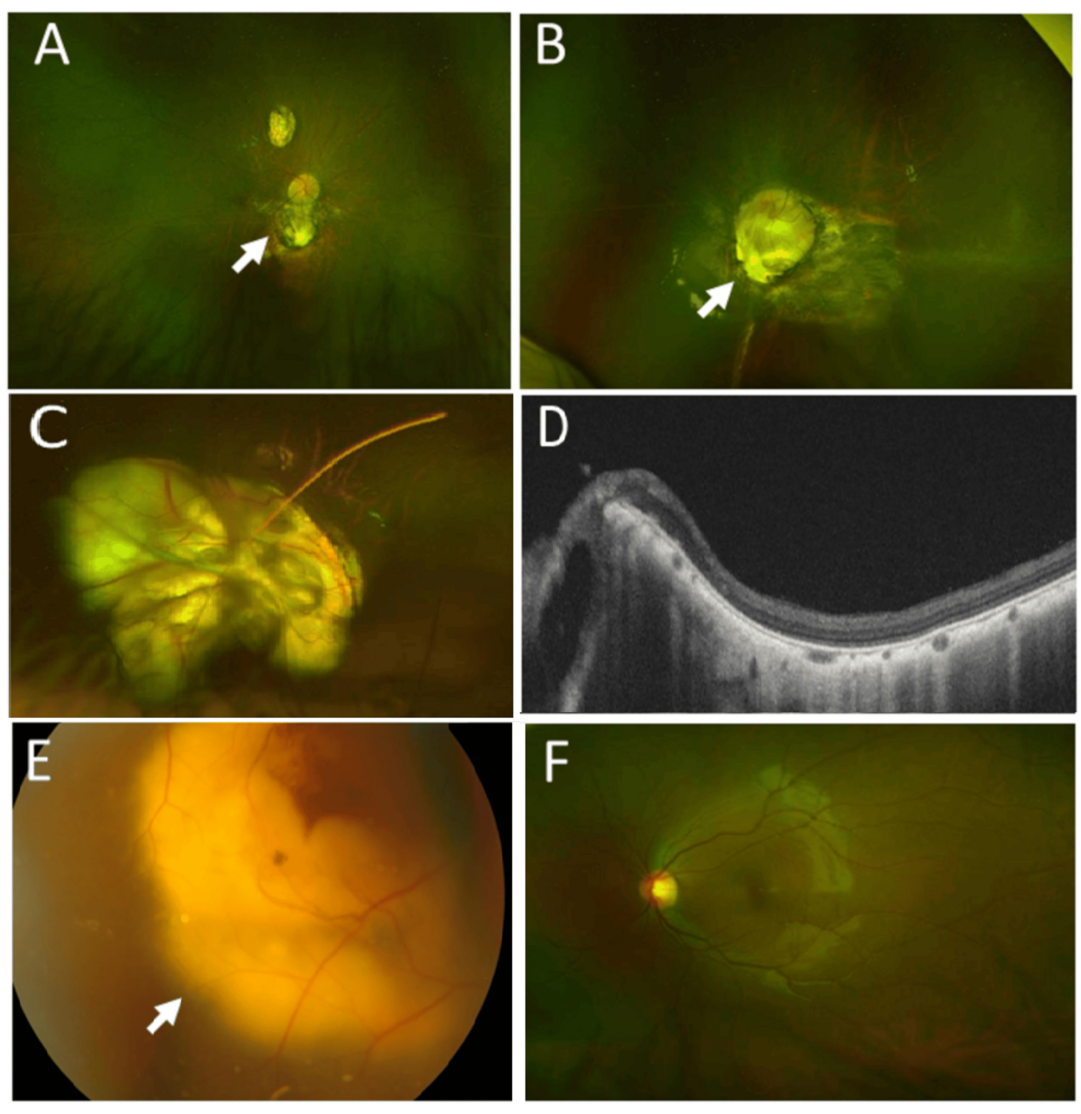
Optic nerve and choroidal coloboma, and Coats disease Case 5: CHARGE syndrome. Widefield fundus photographs, showing choroidal coloboma (arrow) in the right eye (A), and optic nerve and choroidal coloboma (arrow) in the left eye (B) at age 18 years. Case 17: CHARGE syndrome. Widefield fundus photograph, showing optic nerve and choroidal coloboma in the left eye (C), and area of coloboma (leftmost) and normal retinal layer in horizontal section image of optical coherence tomography (D) at age 11 years. Case 7: Coats disease. Fundus photograph in the right eye with yellowish subretinal exudates (arrow) before panretinal photocoagulation at age three years (E). Normal widefield fundus photograph in the left eye at age 17 years (F)

Table 1 summarizes 18 children (11 female and 7 male children). The age at the initial visit ranged from 10 days after birth to 11 years, with a median of one year and four months, and the age at the last visit ranged from 10 to 32 years, with a median of 15 years. The follow-up periods ranged from 10 to 21 years at a median of 15 years. Figures 1-7 show clinical images in each case. The diagnoses were FEVR in eight children (Figures 1-3), PFV in five (Figures 4, 5), morning glory disc anomaly in two (Figure 6, Cases 1 and 2), optic nerve and choroidal coloboma in two (Figures 7A-7D, Cases 5 and 17), and Coats disease in one (Figures 7E, 7F, Case 7). The two children (Cases 5 and 17) with optic nerve and choroidal coloboma in both eyes were diagnosed with CHARGE syndrome in association with bilateral deafness and congenital heart disease. The two children with morning glory disc anomaly (Cases 1 and 2) did not experience any systemic complications.

Full-correction glasses, based on cycloplegic refraction with 1% cyclopentolate, were prescribed in eight children, while the remaining 10 children did not wear glasses. Among nine children with the uncorrected visual acuity of 1.0 or better in one eye and the visual acuity in the other eye ranging from light perception to 0.01, eight children did not wear glasses, and one child (Case 14) wore glasses with hyperopic correction. The diagnoses in these nine children, with uncorrected visual acuity of 1.0 or better in one eye, were PFV in five children, morning glory disc anomaly in two, FEVR in one, and Coats disease in one. In two children with the diagnosis of FEVR who did not wear glasses, one child (Case 3) did not wear glasses because she had mild intellectual disability at the first presentation at the age of 11 years, while another child (Case 13) had only mild myopic astigmatism in the 15-year follow-up.

When one child (Case 14) with hyperopic correction for uncorrected visual acuity of 1.0 in one eye and PFV with light perception in the other eye was excluded, seven children wore full-correction glasses. In these seven children, the best corrected visual acuity in the better eye ranged from 0.2 to 0.9 at a median of 0.5. In contrast, the visual acuity in the other eye ranged from light perception to 0.1 at a median of 0.03. The diagnoses of the seven children were FEVR in five children and CHARGE syndrome in two. The five children with FEVR showed myopic astigmatism in both eyes, while the two children with CHARGE syndrome showed hyperopic astigmatism in both eyes.

Regarding the wearing of full-correction glasses, children with bilateral involvement of congenital eye diseases, such as FEVR and CHARGE syndrome, wore glasses, whereas those with unilateral involvement, such as PFV and morning glory disc anomaly, did not wear glasses. When two categories of FEVR and PFV in a relatively large number were picked up, five of eight children with FEVR wore full-correction glasses, whereas all five children with PFV did not wear glasses (significantly different between the two groups, p = 0.024, chi-square test).

## Discussion

PFV usually occurs in the unilateral eye and often shows accompanying features of a small cornea (microcornea) and a small eye (microphthalmia) [13-16]. The children with PFV show a hyaloid artery remnant, which manifests as a vitreous stalk-like opacity from the optic disc to the posterior surface of the lens to a different extent, varying from individual to individual. Morning glory disc anomalies also occur with no vitreous opacity in the unilateral eye. In contrast, FEVR occurs in bilateral eyes, based on the genetic background, and manifests as different levels of clinical severity between both eyes [9-12]. The clinical manifestations range from mild peripheral retinal degeneration only to a dragged optic disc and to falciform retinal detachment.

Coats disease occurs in the unilateral eye with a congenital aspect and shows retinal vascular abnormalities to a varying extent, which leads to a differing area of involvement with subretinal exudation [25]. Optic nerve coloboma, choroidal coloboma, and also iris coloboma are caused by incomplete closure of the embryonic fissure in the process of the eyeball development and, thus, manifest as a defect of the tissue in the lower part of the eye [17,18,20,21]. These colobomas may occur in the unilateral eye and often occur in bilateral eyes, especially in syndromic cases with a genetic background, such as CHARGE syndrome [26]. All these congenital anomalies of the ocular fundus may present a sign of leukocoria, which indicates a white-color reflex from the ocular fundus instead of a normal red-color reflex, and have to be differentially diagnosed from retinoblastoma as a malignancy. Under the circumstances, the signs of a small cornea and a small eye are directed toward the diagnosis of congenital anomalies.

The present series of 18 children is unique in that the consecutive children with congenital anomalies in the ocular fundus, irrespective of the diagnoses, were altogether reviewed from the standpoint of refractive error correction with glasses. Children with unilateral eye diseases such as PFV, morning glory disc anomalies, and Coats disease had naturally better levels of uncorrected visual acuity in the healthy eyes, and thus, did not have to wear glasses. In contrast, children of bilateral eye involvement with FEVR and CHARGE syndrome usually wore full-correction glasses throughout the course. It should also be noted that children with FEVR showed myopic astigmatism, in contrast with hyperopic astigmatism in two children with CHARGE syndrome.

There is no doubt in the diagnosis of morning glory disc anomaly in Case 1, who showed typical features of funnel-shaped excavation of the optic nerve head and the central glial tufts (Figures 6A, 6B). In contrast, the morning glory disc anomaly was considered more suitable than optic nerve coloboma in Case 2 (Figure 6F), who underwent vitrectomy and cataract surgery with intraocular lens implantation. Indeed, the fundus manifestation in Case 2, with the diagnosis of morning glory disc anomaly, differed from the optic nerve and choroidal coloboma associated with CHARGE syndrome in two children (Figures 7B, 7C, Cases 5 and 17). The Case 8 child developed a mature cataract (Figure 4B) in the follow-up of PFV in the left eye to the final visit at the age of 15 years, but he and his family did not wish to have cataract surgery since the initial visual acuity was poor at 0.02.

A major limitation in this study is the fact that the series of 18 children did not reflect the real-world prevalence of congenital eye diseases since those children were all referred for their diagnosis and management. In the setting of a referral-based hospital, children with mild manifestations of congenital eye diseases would not be referred to a university hospital, but rather would be followed at eye clinics. In contrast, congenital eye diseases in association with systemic diseases would be naturally referred to a university hospital. This style of management would mark two children with CHARGE syndrome in this series of 18 children, even though the syndrome is extremely rare.

The main strength of this study could be the exceptionally long follow-up period, which would provide a unique longitudinal perspective with clinical images as a valuable asset. However, the study might be significantly hampered by its retrospective and descriptive nature and also by a tiny sample size with heterogeneous diseases. The retrospective design would be vulnerable to missing data, and the unexplored impact of confounding variables like cognitive delay or ocular surgeries might be present in this study.

## Conclusions

This study aimed to address the clinical challenges and gaps in the management of children with congenital anomalies of the ocular fundus by retrospectively analyzing long-term visual outcomes, refractive error patterns, and the use or nonuse of full-correction glasses over a follow-up period of at least 10 years. In these conditions, which have often been overlooked due to their rarity and complexity, and where clinical decision-making is frequently inconsistent and poorly guided by the evidence, the author aimed to clarify whether refractive correction via cycloplegic testing could meaningfully contribute to visual development, particularly in cases with asymmetric or bilateral involvement.

In the present study, the 18 consecutive children with congenital anomalies in the ocular fundus, irrespective of the diagnoses, were reviewed from the standpoint of refractive error correction with glasses. Children with unilateral eye anomalies such as PFV and morning glory disc anomaly did not wear glasses since their healthy eyes had excellent levels of uncorrected visual acuity. In contrast, children with bilateral eye involvement due to FEVR and CHARGE syndrome wore full-correction glasses. Although the conclusions might be largely intuitive and already well-established in pediatric ophthalmology practice, the rough information regarding full-correction glasses in each category of the diseases, together with clinical images, would help clinicians cope with rare congenital eye diseases such as FEVR, PFV, and optic disc anomalies such as morning glory disc anomaly and optic nerve coloboma.
